# Microstructure and Mechanical Properties of Modified 316L Stainless Steel Alloy for Biomedical Applications Using Powder Metallurgy

**DOI:** 10.3390/ma15082822

**Published:** 2022-04-12

**Authors:** Sadaqat Ali, Muhammad Irfan, Usama Muhammad Niazi, Ahmad Majdi Abdul Rani, Ahmad Rashedi, Saifur Rahman, Muhammad Kamal Asif Khan, Mabkhoot A. Alsaiari, Stanislaw Legutko, Jana Petrů, Antonin Trefil

**Affiliations:** 1 School of Mechanical & Manufacturing Engineering, National University of Sciences and Technology (NUST), H-12, Islamabad 44000, Pakistan; 2 Electrical Engineering Department, College of Engineering, Najran University Saudi Arabia, Najran 61441, Saudi Arabia; miditta@nu.edu.sa (M.I.); srrahman@nu.edu.sa (S.R.); 3 Mechanical Engineering Technology Department, National Skills University, Islamabad 44000, Pakistan; 4 Mechanical Engineering Department, Universiti Teknologi PETRONAS (UTP), Seri Iskandar 31750, Malaysia; majdi@utp.edu.my; 5 School of Mechanical & Aerospace Engineering, Nanyang Technological University, 50 Nanyang Avenue, Singapore 639798, Singapore; amma0002@e.ntu.edu.sg; 6 Mechanical Engineering Department, College of Engineering, Najran University, Najran 61441, Saudi Arabia; mkkhan@nu.edu.sa; 7 Empty Quarter Research Unit, Chemistry Department, College of Science and Art at Sharurah, Promising Centre for Sensors and Electronic Devices (PCSED), Najran University Saudi Arabia, Najran 61441, Saudi Arabia; mabkhoot.alsaiari@gmail.com; 8 Faculty of Mechanical Engineering, Poznan University of Technology, 60-965 Poznan, Poland; stanislaw.legutko@put.poznan.pl; 9 Faculty of Mechanical Engineering, VSB—Technical University of Ostrava, 708 00 Ostrava, Czech Republic; jana.petru@vsb.cz (J.P.); antonin.trefil@vsb.cz (A.T.)

**Keywords:** AISI 316L stainless steel, mechanical properties, microhardness, tensile strength, compressive strength

## Abstract

AISI 316L stainless steel (SS) is one of the extensively used biomaterials to produce implants and medical devices. It provides a low-cost solution with ample mechanical properties, corrosion resistance, and biocompatibility compared to its counterpart materials. However, the implants made of this material are subjected to a short life span in human physiological conditions leading to the leaching of metal ions, thus limiting its use as a biomaterial. In this research, the addition of boron, titanium, and niobium with varying concentrations in the SS matrix has been explored. This paper explores the impact of material composition on modified SS alloy’s physical and mechanical properties. The study’s outcomes specify that the microhardness increases for all the alloy compositions, with a maximum increase of 64.68% for the 2 wt.% niobium added SS alloy. On the other hand, the tensile strength decreased to 297.40 MPa for the alloy containing 0.25 wt.% boron and 2 wt.% titanium additions compared to a tensile strength of 572.50 MPa for pure SS. The compression strength increased from 776 MPa for pure SS to 1408 MPa for the alloy containing niobium and titanium additions in equal concentrations.

## 1. Introduction

Biomedical implants and devices are extremely valuable in improving human quality of life, nature, and lifespan [1,2]. The development of new biomaterials with specific physical and mechanical properties is intimately linked to the evolution of implant manufacturing [3,4]. Since the inaugural summit on biomaterials development in 1969, this discipline has received much attention throughout the last few decades [5]. Since then, a continuous effort has been made to create novel materials and improve implant and implant manufacturing procedures [6]. In this perspective, choosing the right biomaterial for implant production is critical for its long-term success in mechanical attributes, biocompatibility, wear resistance, corrosion resistance, cost, and ease of manufacture [7,8]. These materials are anticipated to convey superior results in their application and usage by performing well within the human body, especially while in contact with body fluids [8,9].

Among the available classes of biomaterials, metallic materials are extensively used, accounting for over 70% of all implants and medical devices [9,10,11]. The selection of metal as an implant material can be attributed to its superior corrosion resistance and mechanical strength required for better performance in long-term implantation [12,13]. The foremost commonly utilized biomaterials include Ti6Al4V, CoCrMo, and AISI 316L stainless steel [14]. Owing to its low cost, reasonable corrosion resistance, and ease of manufacturing, one of the most frequently used biomaterials in the manufacturing of implants is austenitic 316L stainless steel, the commercially available biomaterial [15,16]. The implants manufactured from this material are cheaper than titanium and cobalt-based alloys by one-tenth to one-fifth [17,18]. The stainless steels contain chromium with a minimum percentage of 11 wt.%, which helps the material from corrosion in severe environments [19]. The increased corrosion resistance of stainless steels is achieved by the presence of nickel, which helps stabilize the austenite formation of iron. On the other hand, this material is prone to localized corrosion attacks. It erodes in human physiological conditions in long-term applications, restricting its use as an implant material [20,21]. In vitro corrosion of stainless steel results in the release of metal ions. The immunological response is inhibited, and the expression of lymphocyte-surface antigens is altered due to the ion release [22,23]. Nickel, iron, and chromium ions accumulate in the tissues around the implant as corrosion products [24,25]. Among released elements, nickel has been found accountable for genotoxic and mutagenic activities, cancer, eczema, and skin itching [26,27]. Despite several efforts on improving the material properties, the premature implant failure and leaching of metal ions from the base material remains unsolved. Some recent articles on implant failure have critically analyzed to determine the cause of loss of implants and implant materials [28,29,30,31,32]. The analysis indicated that the implant failures were due to several factors: poor material quality, inferior material surface finishing, cracks initiation from implant fabrication or coating method, and high wear and corrosion rate of the implant material. The recently failed implants and implant materials lead to the conclusion that optimizing the processing parameters of the biomaterials has great potential in improving the material properties.

As a result of these constraints, scientists are working on producing new materials. Furthermore, various surface modification approaches are being investigated to combat implant erosion and improve corrosion resistance. These methods should be able to handle the desired biological response at the interface between human tissue and implant material with ease. Chemical, mechanical, and physical surface modification approaches are researchers’ recommended techniques [10,33,34]. Low coating adhesion strength, non-uniformity, fracture development, high cost, amorphous coating generation, and inability to coat complex forms are shortcomings of these surface modification techniques [35,36,37].

Powder metallurgy (PM) allows simultaneous surface modification and processing of new biomaterials, which can be used to investigate alternative cost-effective production methods for implant material [38,39]. Using this technology, a modified stainless steel alloy with a protective surface layer is necessary to tackle the concerns outlined above. To increase the performance of this material, the behaviour of simultaneous additions of boron, titanium, and niobium in stainless steel alloy can be examined. For producing a protective surface layer, consolidation factors such as compaction pressure, sintering temperature, and dwell time have not been explored in our previous studies [15,25,38,40,41,42,43]. This research work examines the effect of material composition on the physical and mechanical properties of modified 316L stainless steel alloy.

## 2. Materials and Methods

This study reinforced the stainless steel matrix with boron, titanium, and niobium additions. Titanium and niobium were added in varying concentrations of 0.5–2 wt.%. Five types of formulations were devised for the development of a customized stainless steel alloy. The first formulation was pure 316L stainless steel as-received powder. The particle size distribution of the as-received 316L stainless steel powder is characterized and presented in Table 1.

The next three formulations contained boron, whereas the last formulation was prepared without adding any boron. Since the boron addition was added for activated sintering, its concentration was fixed at 0.25 wt.% for all boron-containing compositions for better sintering results. These prepared formulations were named pure 316L SS, boron-titanium admixed 316L SS (B-Ti 316L SS), boron-niobium admixed 316L SS (B-Nb 316L SS), boron-titanium-niobium admixed 316L SS (B-Ti-Nb 316L SS), and titanium-niobium admixed 316L SS (Ti-Nb 316L SS). These five formulations are depicted in Table 2. The impact of different powder concentrations on the final alloy system was studied regarding sintered density, corrosion resistance, and in vitro cytotoxicity assessment. These results are already published in our previous papers.

**Figure 1:**
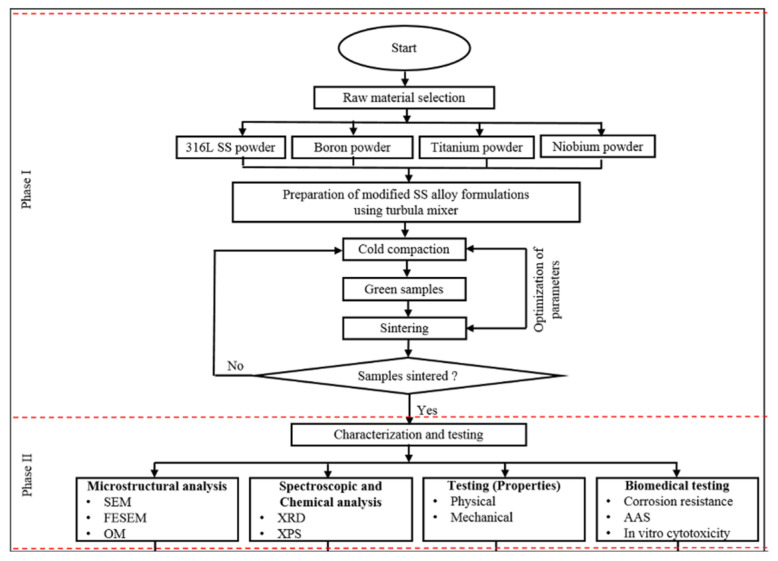
Flow diagram of the whole research methodology.

Using a Turbula mixer (Malaysia), each formulation was created by combining the appropriate amount of each powder. The powders of each formulation were then compacted into a disc shape using a uniaxial cold compaction process. The steel dies utilized in this research produced samples of 30 mm diameter and 5 mm thickness compacts. After that, the green compacts were sintered at 1200 °C in a nitrogen environment. The schematic of the process is shown in Figure 1.

Phase I of the flow diagram shows the preparation of the formulations and the preparation of samples. In contrast, phase II represents the characterization and testing of the prepared samples. This paper mainly focuses on testing the physical and mechanical properties, including microhardness, tensile strength, and compressive strength.

The green density of compacted samples indicates the compressibility of metal powder at a given compaction pressure. It depends on the chemical composition, particle shape, and particle density. Firstly, the green densities of compacted samples were determined by measuring the thickness and diameter of the samples using a geometric technique. The values of green densities were determined by dividing each sample’s mass by its volume. Secondly, the sintered density of each sample was calculated using Archimedes’ Principle via the standard test method (ASTM B962-14). The density was measured using an HR-150 AZ analytical balance. Each formulation’s sintered density was measured in three samples, and the average value was recorded.

A Vickers hardness tester was utilized to determine the microhardness of all formulations. All the compositions were subjected to a 1.96 N force with a 15-s duration. At least five values from various positions on the test samples were recorded for each sample, with the averaged values generated and reported.

**Figure 2:**
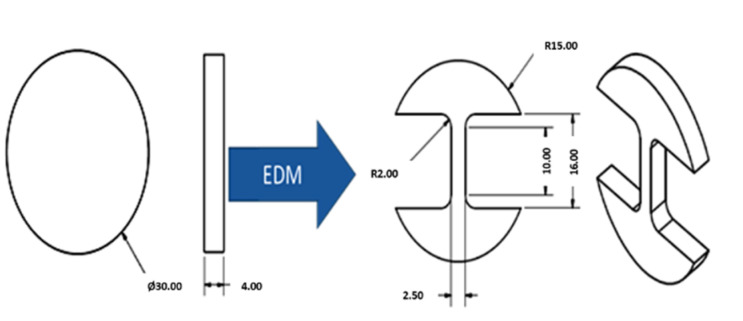
Schematic diagram of tensile test sample produced by EDM.

**Figure 3:**
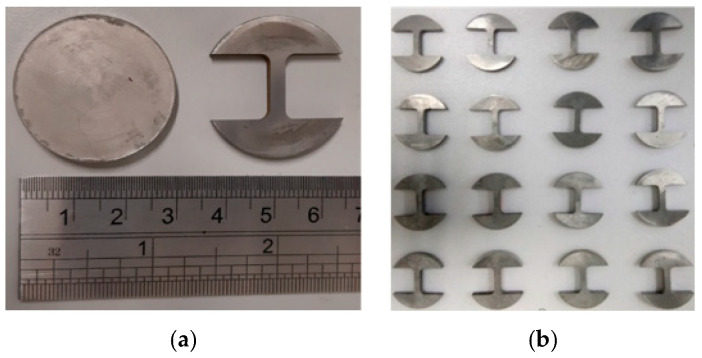
EDM machined samples for tensile testing (**a**) original sample before cutting (**b**) ready samples for tensile testing.

The tensile test samples were cut from the disc shape samples corresponding to ASTM standard B925-08 (E8/E8M–13a) for tensile strength testing. Electro-discharge machining (Swiss Wire EDM, Costa Mesa, CA, USA) was used to cut the samples according to the standard with a gauge section of 2.5 mm (width) × 10 mm (length). This approach of sample extraction via EDM from disc-shaped samples for tensile testing was also practiced by other researchers. The schematic drawing with dimensions for the sample fabrication and EDM samples are shown in Figure 2 and Figure 3. Figure 3a shows the sample from which the tensile samples were prepared, whereas Figure 3b presents the tensile test samples ready for testing. The tensile testing was conducted at a 0.01 mm/min strain rate using a Shimadzu universal tensile testing machine (Shimadzu, Colombia, MD, USA).

**Figure 4:**
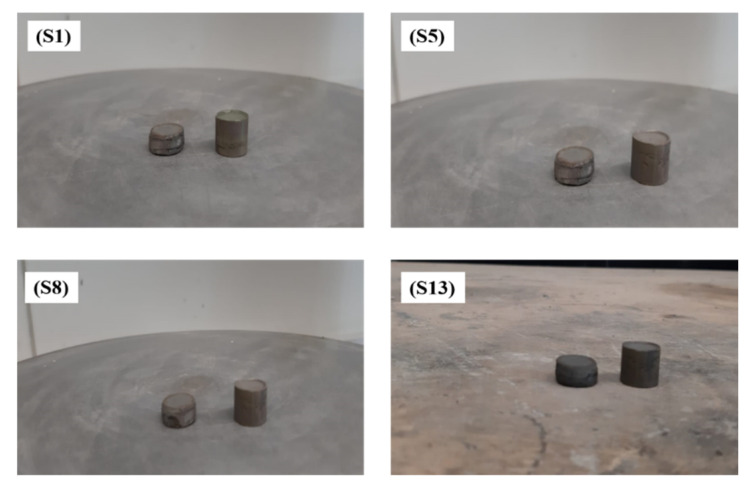
Test samples before and after compression testing.

The compressive strength of the developed alloy systems was found by a compressing test of cylindrical shape samples (13 mm diameter and 25 mm in length) as shown in Figure 4. These samples were also fabricated by following the procedure mentioned above of compaction and sintering processing. The tests were done via an Amsler 100 kN (Zwick/Roell) testing system with a crosshead speed of 0.5 mm/min equipped with 9600 series software (Kennesaw, GA, USA) by following the ASTM E9-09 standard procedures. The compressive strength was recorded for all samples tested under compression testing.

## 3. Results and Discussion

All the formulations’ sintered samples were examined for their physical and mechanical properties. The properties studied in this research work include microhardness, tensile and compressive strength. The results of each property have been discussed in the following sections.

### 3.1. Characterization of Mixed Proportions

**Figure 5:**
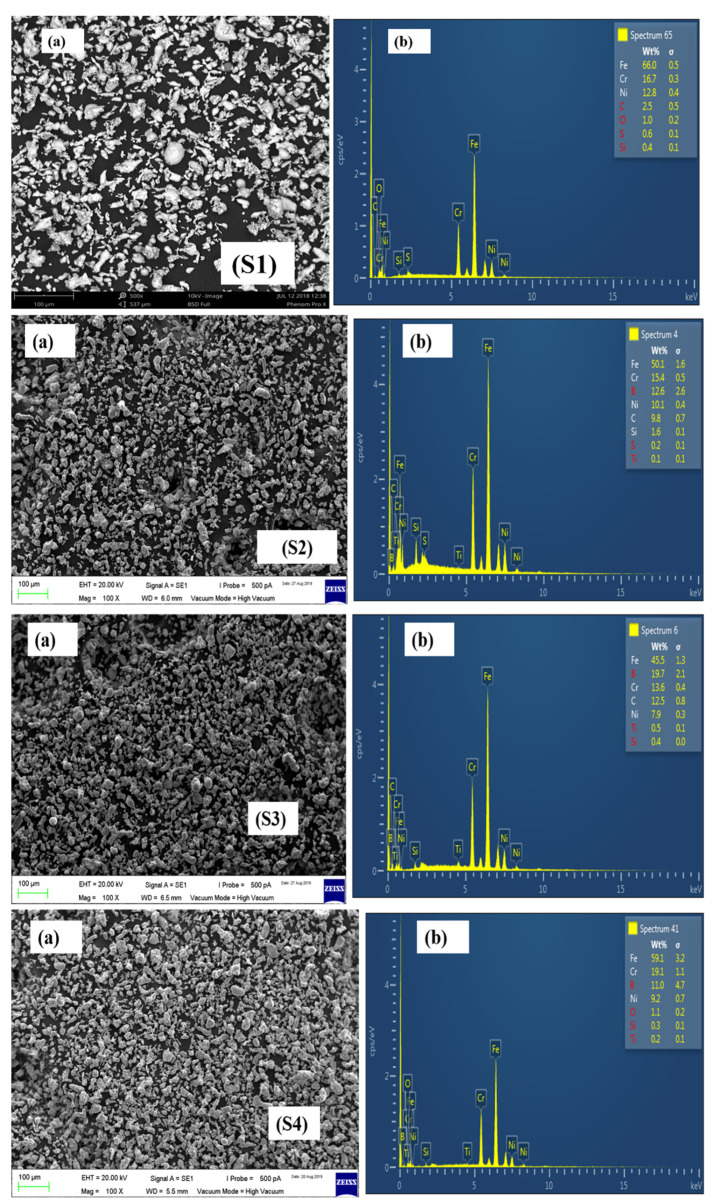
SEM images of S1, S2, S3, and S4 powder formulations.

In this research work, five different formulations were designed, the details of which have already been discussed in Table 1. The developed formulations were investigated for their homogeneity by SEM and EDX analysis. The SEM and EDX analysis of boron-titanium added 316L stainless steel formulations is presented in Figure 5.

The SEM analysis revealed uniform dispersion of the powders. The presence of boron and titanium particles was confirmed from the EDX results. The respective concentrations of each constituent at a particular spectrum were given by EDX and reported for all the powder formulations.

**Figure 6:**
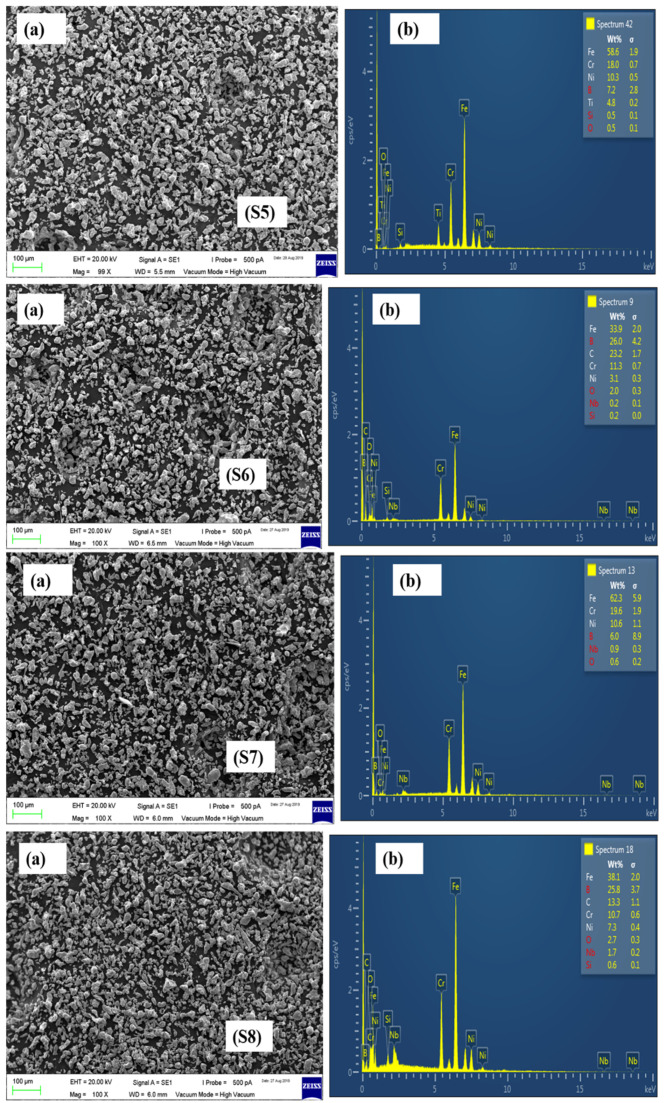
SEM images of S5, S6, S7, and S8 powder formulations.

**Figure 7:**
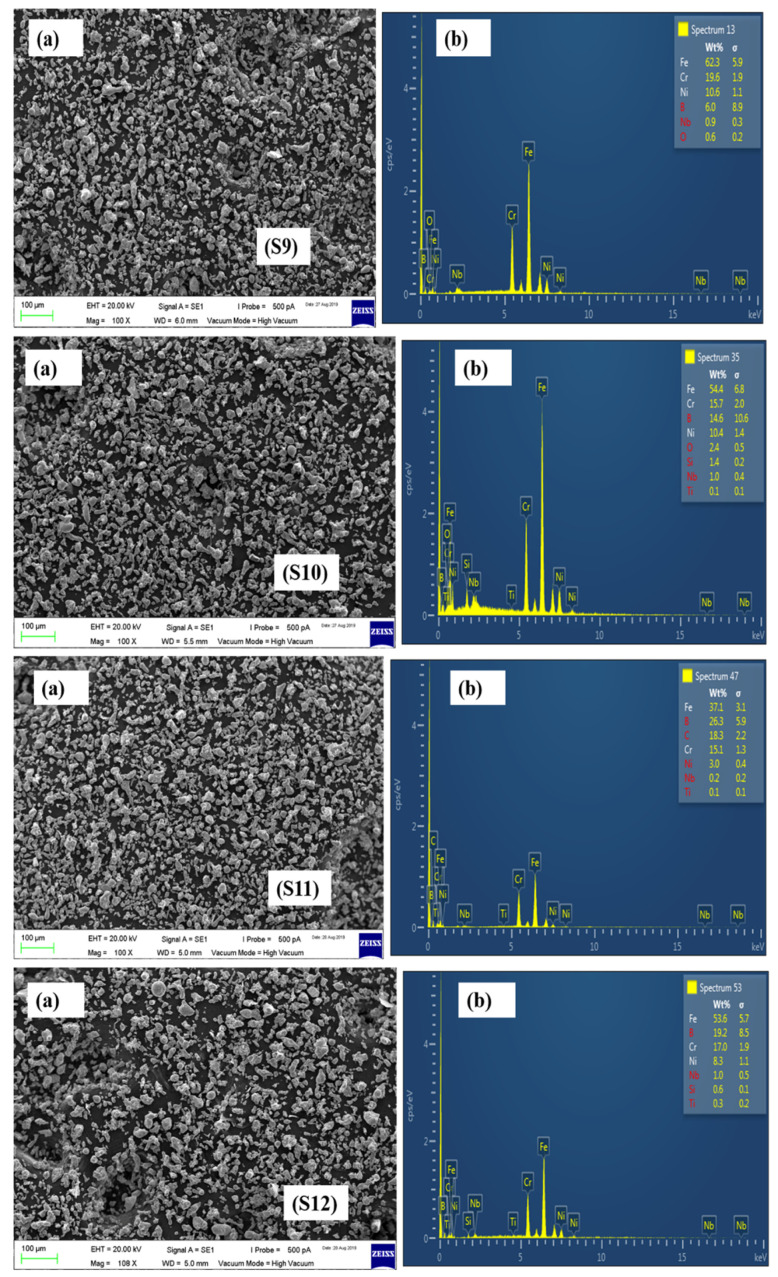
SEM images of S9, S10, S11, and S12 powder formulations.

**Figure 8:**
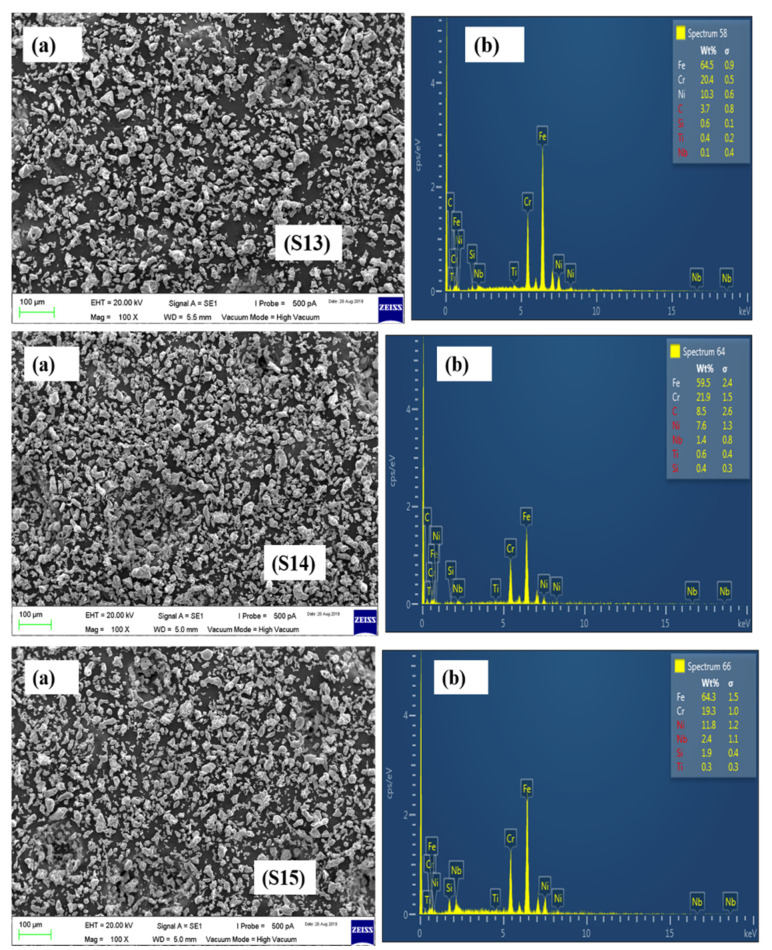
SEM images of S13, S14, and S15 powder formulations.

The SEM and EDX results of the remaining formulations have been presented in Figure 6, Figure 7 and Figure 8.

### 3.2. Density Measurement

Using the geometric method described in the previous section, the green density of pure 316L stainless steel was obtained to be 6.5 g/cm^3^. The sintered density was observed to be 7.575 g/cm^3^, and the relative density was found to be 95.88%. This was the maximum value among all the compositions studied in this research, as reported in Table 3.

The boron-titanium admixed 316L stainless steel formulations were also compressed and sintered using the procedures discussed. The green, sintered, and relative densities of the B-Ti 316L SS samples were calculated and tabulated in Table 3. As the amount of titanium added increased, the green density values decreased. The dispersion of boron and titanium in the matrix caused a drop in the green density value. Furthermore, titanium had lower density than stainless steel, lowering the green density. Relative densification was improved because of the sintering environment and dwell time duration. Relative densification of 94.40% was noted for S2 samples containing 0.5 wt.% titanium, whereas close densification of 89.14% was seen for 2 wt.% titanium-added samples. The inclusion of boron improved the densification process significantly, as reported in the literature [44,45,46]. Its addition kept the sintered density of samples close to that of pure 316L stainless steel samples.

The green, sintered, and relative densities of the boron-niobium-added samples produced have been depicted in Table 3. The green density of the SS samples with boron and niobium addition decreased slightly compared to the green density of pure 316L stainless steel samples. It was because boron and niobium particles attempted to penetrate the stainless steel matrix. Sintering the samples in a nitrogen environment enhanced the sintered density. The results demonstrate that the sintering environment and temperature were favourable for appropriate densification. The density of the sintered samples with boron and niobium additions led to a reduced sintered density. Although the inclusion of boron and niobium reduced the sintered density of the samples, their impact on hardness, corrosion resistance, and cytotoxicity was apparent.

The boron-titanium-niobium admixed 316L SS (B-Ti-Nb 316L SS) formulations were prepared by the same method as discussed in the previous sections. The green, sintered, and relative densities of the boron-titanium-niobium-added samples produced are depicted in Table 3. When comparing the green density of boron-titanium-niobium-added stainless steel samples to the green density of pure 316L stainless steel samples, the density of boron-titanium-niobium-added stainless steel was lower. The sintered samples’ densification was improved because of the sintering environment and dwell time duration. Relative densification of 92.14%, 91.79%, and 91.66% was observed for samples S10, S11, and S12, respectively.

The titanium-niobium admixed 316L stainless steel formulations were prepared using the procedures discussed in previous sections. The compositions without boron addition had lower sintered densities than the boron-added samples. This indicates that the boron aided in the densification process by forming a liquid phase because of the eutectic reaction between boron and iron. This reaction occurred at a higher temperature than that required for eutectic transition. At this point, a liquid phase arose, which aided in better sintering by minimizing the porosity. Boron segregated by producing a layer on grain boundaries, providing a high diffusivity rate which aided in rapid powder densification. The green, sintered, and relative densities of the Ti-Nb 316L SS samples were calculated and are tabulated in Table 3.

### 3.3. Optical Microscopy of Sintered Samples

**Figure 9:**
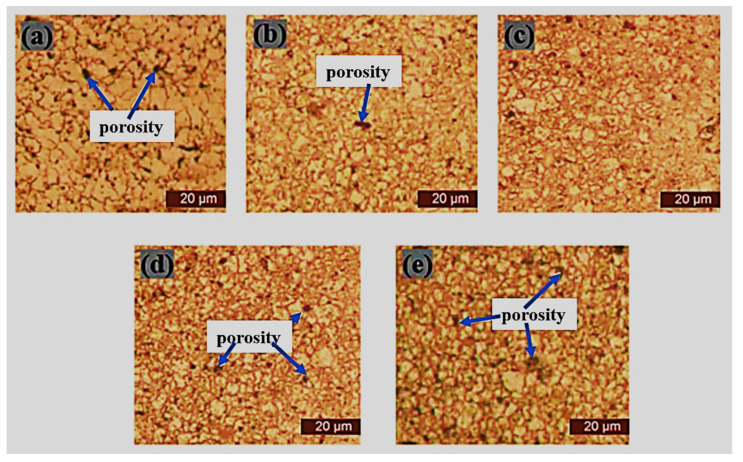
Microstructure of sintered samples (**a**) S1, (**b**) S2, (**c**) S3, (**d**) S4, and (**e**) S5.

**Figure 10:**
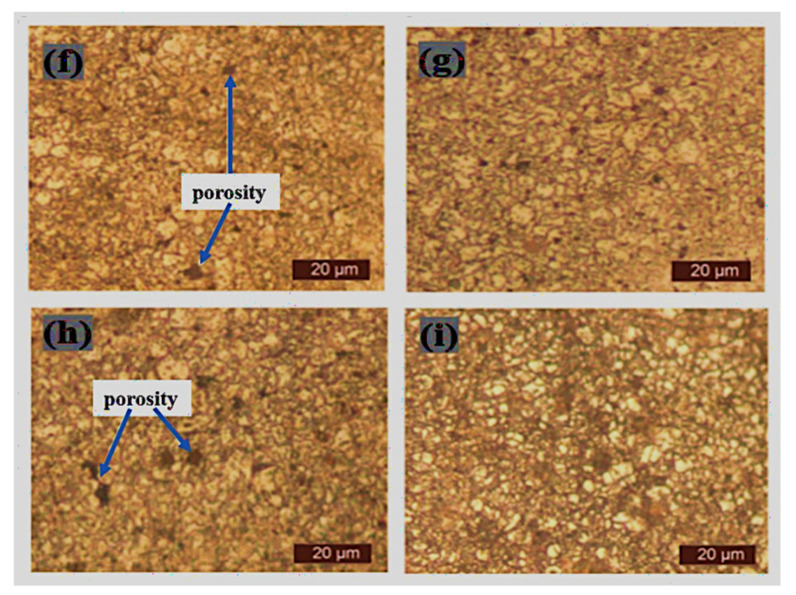
Microstructure of sintered samples (**f**) S6, (**g**) S7, (**h**) S8, and (**i**) S9.

**Figure 11:**
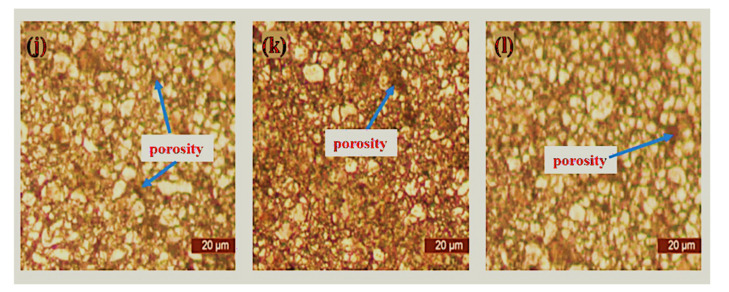
Microstructure of sintered samples (**j**) S10, (**k**) S11, and (**l**) S12.

**Figure 12:**
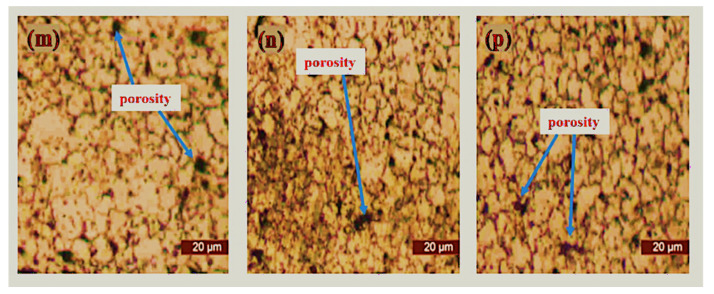
Microstructure of sintered samples (**m**) S13, (**n**) S14, and (**p**) S15.

The microstructural characterization of the sintered samples was carried out via optical microscopy (Toronto, ON, Canada) after complete metallographic preparation. The micrographs of the sintered samples as viewed from an optical microscope are depicted in Figure 9, Figure 10, Figure 11 and Figure 12. It was observed that pure 316L stainless steel sintered at 1200 °C temperature had a dense structure without significant pores and with clear grain boundaries. Furthermore, boron, titanium, and niobium additions did not provide a barrier during the sintering process with significantly low porosity.

Figure 9 shows the microstructure of pure 316L stainless steel and boron-titanium admixed stainless steel as observed under an optical microscope. The addition of reinforcements did not hinder the densification process, and clear boundaries could be visualized. There were fewer pores present in the samples and pointed out in the respective Figures. Figure 10 presents the microstructure for boron-niobium admixed stainless steel samples. The grain boundaries suggest that the niobium did not hinder the sintering process, and adequate densification was observed for all the boron-niobium-containing samples. Figure 11 shows the microstructure for boron-titanium-niobium admixed stainless steel samples. The micrographs indicate the densification of these samples with clear grain formation. The porosities present in the samples have been highlighted accordingly. Figure 12 shows the microstructure for titanium-niobium admixed stainless steel. These samples did not have boron and only contained titanium and niobium as the reinforcements. More pores as compared to other compositions could be noticed in these sample compositions.

### 3.4. Microhardness of Sintered Samples

The microhardness of sintered samples is given in Table 4. Pure 316L stainless steel samples showed a microhardness of 235 HV.

The microhardness of (B-Ti 316L SS) formulations increased the samples’ hardness compared to that of pure 316L stainless steel samples. From Table 4, it should be noted that as the titanium concentration increased, so did the microhardness. An enhancement of microhardness was noted for all the samples. The samples with 2 wt.% titanium additions had a microhardness of 366 HV.

The infusion of nitrogen into the matrix increased the microhardness of the (B-Nb 316L SS) formulations’ samples, indicating an increase in microhardness. The inclusion of boron contributed significantly to the increase in microhardness. The addition of niobium also favored an upsurge in microhardness. The quantity of niobium addition also influenced the microhardness, and it increased with increasing niobium quantity. For samples with a 2 wt.% niobium addition, a maximum of 387 HV was found. The microhardness of all the samples of this formulation is shown in Table 4.

The microhardness of B-Ti-Nb 316L SS formulations is shown in Table 4. The microhardness of these formulations was closely related to each other. A maximum of 385 HV was demonstrated for the S12 sample. The microhardness values for S10 and S11 were 380 and 376 HV, respectively. The boron addition in these samples impacted their microhardness values and was higher than in the formulations without boron addition. This indicates that boron helped increase the microhardness of the sintered samples when alloyed in stainless steel.

The microhardness of Ti-Nb 316L SS formulations was found using the same technique as discussed previously. These formulations had lower microhardness values as compared to those of the ones containing boron. Table 4 shows that the S15 sample had a maximum microhardness of 350 HV.

There was a positive correlation observed for the microhardness of all the samples by increasing the reinforcements contents compared to pure 316L stainless steel samples. The microhardness was dependent on the localized deformation; hence the inclusion of rigid reinforcements tended to raise the resistance to localized plastic deformation. Moreover, since the sintering time was 8 h, the extra nitride layer deposited on the material surface led to the increase in microhardness of the test samples.

### 3.5. Tensile Strength and Fracture Analysis

The tensile testing machine was utilized to determine the tensile strength of all the formulations by applying a tensile load. The ductility (percentage of elongation) and ultimate tensile strength (UTS) were calculated and discussed.

Tensile testing of a pure 316L stainless steel sample was performed in accordance with ASTM standards, and the results are listed in Table 5. The UTS of pure 316L stainless steel was determined to be 540.7 MPa. It was the greatest of all compositions. The sample’s percentage elongation was 21.90% which was the greatest of all the samples.

The tensile testing of B-Ti 316L SS formulations was conducted to explore the impact of material composition on the tensile strength of sintered samples, and the results are tabulated in Table 5. The findings reveal a significant decrease in tensile strength with the addition of boron and titanium. The UTS decreased with increasing titanium contents. The results show that the addition of titanium reduced the tensile strength of stainless steel. Similar results of decrease in tensile strength by the addition of titanium boride have been reported by Sulima et al. [47]. A maximum value of 358.91 MPa of UTS was observed for samples containing 0.5 wt.% titanium addition, its value decreased to 304.44 MPa for 2 wt.% titanium-added samples. The percentage elongation was lower than that of pure 316L stainless steel samples and was between 10.04 and 10.41%.

The tensile testing of B-Nb 316L SS formulations showed a similar trend, and a decrease in UTS was observed for the samples. The results are tabulated in Table 5. They indicate that the boron and niobium addition decreased the UTS from 540.7 MPa for pure 316L stainless steel samples to 449.84 MPa for 0.5 wt.% niobium-added samples. It can be noted that the UTS value decreased with increasing niobium content. The lowest value of 413.45 was observed for samples containing 2 wt.% niobium. The percentage elongation for the samples remained lower than that of pure 316L stainless steel samples and remained between 14.15 to 17.02%. The tensile strength of these formulations was better than the tensile strength of titanium-added samples with similar content values when alloyed to 316L stainless steel. Moreover, the hardness values of boron-added samples were better than the hardness values of titanium-added samples.

The tensile testing of B-Ti-Nb 316L SS formulations was conducted to examine the influence of material composition on tensile strength. The results of the testing are tabulated in Table 5. The results show a decrease in tensile strength by adding boron, titanium, and niobium in 316L stainless steel. The UTS decreased with increasing titanium contents, and the results of S11 and S12 were like the tensile strength of boron-titanium-added samples. The UTS of S10 was better among the other two samples and was near the UTS of sample S9, containing the 2 wt.% boron-niobium sample.

The tensile strength of Ti-Nb 316L SS compositions was better than that of the boron-containing samples with similar concentrations of titanium and niobium. The results indicate that although boron increased the microhardness of the sintered samples, it caused the tensile strength of the material to deteriorate. The findings also show that UTS with a higher niobium concentration had better tensile strength than UTS with titanium addition. For the S13 sample, a maximum UTS of 438.68 MPa was assessed, and the lowest was found for the S14 composition. The results of the testing are shown in Table 5.

**Figure 13:**
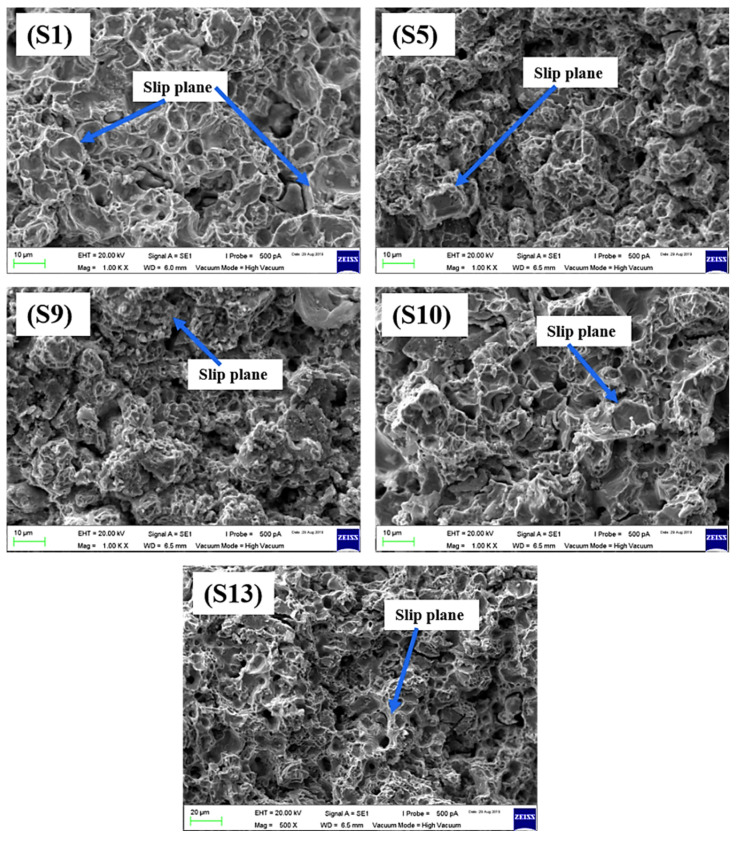
SEM images of tensile fracture surfaces.

The SEM analysis of fractured tensile test samples was conducted to observe the fracture surface. Figure 13 illustrates the SEM images of selected fractured samples. The figure indicates that nearly all the samples showed good sinterability with no significant porosity. The fracture morphologies revealed the uniform dispersion of boron, titanium, and niobium additions. There were no visible agglomerates in the fractured sample surfaces, indicating that the powders were uniformly mixed in their respective ratios. The mixing time of 8 h was adequate to uniformly mix the proportions and disperse the additives uniformly throughout the stainless steel matrix.

### 3.6. Compression Testing of Sintered Samples

The compression testing of all the powder formulations sintered samples was conducted to study the effect of material composition on compressive yield strength. For this purpose, compressive samples were prepared in a die according to the ASTM standard E9–09. The compressive testing was carried out on a 200 kN capacity UTM machine (Shimadzu, Colombia, MD, USA). All the samples were compressed using 200 kN force to calculate the deformation and yield strength of compressive samples. The samples experienced an approximately one-half reduction in size without any breakage. The test samples before and after compression testing are shown in Figure 3.

The pure 316L stainless steel samples were compressed at 200 kN without any material breakage, as shown in Figure 3a. The compressed samples at 200 kN force showed a yield strength of 776 MPa.

The compressive strength of boron-titanium-added 316L stainless steel samples was calculated using the same procedure. The samples were compressed to 200 kN force without any breakage of the samples. The yield strength of all these samples is given in Table 6. The results indicate that the yield strength increased with increasing titanium content, and a maximum of 987 MPa yield strength was observed for the samples containing 2 wt.% titanium additions.

The boron-niobium-added 316L stainless steel samples were compressed at 200 kN without any material breakage. The yield strength of all these samples is given in Table 6. The results indicated a drastic increase in yield strength with increasing niobium content. Maximum yield strength of 1318 MPa was observed for sample S9 containing 2 wt.% niobium additions.

The compressive strength of boron-titanium-niobium-added 316L stainless steel samples was calculated using the same procedure. The samples were compressed to 200 kN force without any breakage of the samples. The yield strengths of all these samples are given in Table 6. The findings show that maximum yield strength of 893 MPa was observed for sample S11. The lowest yield strength was demonstrated by S12, which was 747 MPa.

The titanium-niobium-added 316L stainless steel samples were compressed at 200 kN without any material breakage. The yield strengths of all these samples are given in Table 6. The findings imply that the yield strength values were almost identical to one another. A maximum yield strength of 1408 MPa was observed for sample S14.

## 4. Conclusions

This research was carried out to synthesize and develop a modified 316L stainless steel alloy by addressing the challenges faced by this material, including poor physical and mechanical properties. A modified 316L stainless steel was developed, and the effects of powder additions were investigated. The five sets of formulations were tested for their suitability by different techniques, and the results were compared. It was concluded that material composition gave a notable impression on the properties of the material.

The elemental boron addition favored the sintering cycle of the 316L stainless steel alloy by increasing the densification process. Maximum densification of 94.40% was observed for the alloy system if 0.25 wt.% boron was pre-alloyed in 316L stainless steel. The addition of boron in the boron-containing compositions helped maintain the densification of the sintered samples near the sintered density of pure 316L stainless steel. The addition of titanium and niobium in their respective formulations helped improve the material’s properties. The microhardness values were increased by pre-alloying titanium and niobium additions. The microhardness of the 2 wt.% boron-niobium-added stainless steel exhibited an increase of microhardness value by 64.68% compared to that of pure 316L stainless steel. The 2 wt.% boron-titanium-added stainless steel samples showed an increase in microhardness value by 55.74%. The tensile strength of 316L stainless steel decreased by alloying with titanium and niobium additions. This could be because the increase of microhardness values led to the increase in brittleness of the material. Thus, the ductility of the material was reduced, leading to a decrease in the tensile strength of the material. The compressive strength, however, increased with the addition of additives. An increase of 69.93% in the compressive yield strength was observed for samples containing 1.5 wt.% niobium addition.

## Figures and Tables

**Table 1:** Particle size distribution of as-received 316L stainless steel powder.

Particle Distribution	D10	D50	D90
Particle Size (µm)	3.98	10.27	19.61

**Table 2:** Composition of the formulations.

S. No	Alloy	Composition
1	S1	Pure 316L stainless steel
2	S2	316L SS + 0.25 B + 0.5 Ti
3	S3	316L SS + 0.25 B + 1 Ti
4	S4	316L SS + 0.25 B + 1.5 Ti
5	S5	316L SS + 0.25 B + 2 Ti
6	S6	316L SS + 0.25 B + 0.5 Nb
7	S7	316L SS + 0.25 B + 1 Nb
8	S8	316L SS + 0.25 B + 1.5 Nb
9	S9	316L SS + 0.25 B + 2 Nb
10	S10	316L SS + 0.25 B + 0.5 Ti + 1.5 Nb
11	S11	316L SS + 0.25 B + 1 Ti + 1 Nb
12	S12	316L SS + 0.25 B + 1.5 Ti + 0.5 Nb
13	S13	316L SS + 0.5 Ti + 1.5 Nb
14	S14	316L SS + 1 Ti + 1 Nb
15	S15	316L SS + 1.5 Ti + 0.5 Nb

**Table 3:** Green, sintered, and relative densities for all formulations.

Alloy	Composition	Theoretical Density (g/cm^3^)	Green Density (g/cm^3^)	Sintered Density (g/cm^3^)	Relative Density (%)
S1	Pure 316L stainless steel	7.90	6.500	7.575	95.88
S2	316LSS + 0.25 B + 0.5 Ti	7.825	6.385	7.387	94.40
S3	316LSS + 0.25 B + 1 Ti	7.796	6.212	7.139	91.57
S4	316LSS + 0.25 B + 1.5 Ti	7.767	6.116	7.008	90.22
S5	316LSS + 0.25 B + 2 Ti	7.739	6.002	6.899	89.14
S6	316LSS + 0.25 B + 0.5 Nb	7.857	6.370	7.411	94.32
S7	316LSS + 0.25 B + 1 Nb	7.860	6.240	7.367	93.72
S8	316LSS + 0.25 B + 1.5 Nb	7.864	6.160	7.285	92.63
S9	316LSS + 0.25 B + 2 Nb	7.867	6.080	7.190	91.39
S10	316L SS + 0.25B + 0.5 Ti + 1.5 Nb	7.770	6.189	7.160	92.14
S11	316L SS + 0.25B + 1 Ti + 1 Nb	7.802	6.194	7.162	91.79
S12	316L SS + 0.25B + 1.5 Ti + 0.5 Nb	7.834	6.086	7.181	91.66
S13	316L SS + 0.5 Ti + 1.5 Nb	7.886	6.192	7.197	91.26
S14	316L SS + 1 Ti + 1 Nb	7.864	6.196	7.134	90.71
S15	316L SS + 1.5 Ti + 0.5 Nb	7.842	6.108	7.126	90.86

**Table 4:** Microhardness of all formulations.

Alloy	Composition	Microhardness
S1	Pure 316L stainless steel	235 HV
S2	316L SS + 0.25 B + 0.5 Ti	286 HV
S3	316L SS + 0.25 B + 1 Ti	318 HV
S4	316L SS + 0.25 B + 1.5 Ti	356 HV
S5	316L SS + 0.25 B + 2 Ti	366 HV
S6	316L SS + 0.25 B + 0.5 Nb	283 HV
S7	316L SS + 0.25 B + 1 Nb	321 HV
S8	316L SS + 0.25 B + 1.5 Nb	360 HV
S9	316L SS + 0.25 B + 2 Nb	387 HV
S10	316L SS + 0.25 B + 0.5 Ti + 1.5 Nb	380 HV
S11	316L SS + 0.25 B + 1 Ti + 1 Nb	376 HV
S12	316L SS + 0.25 B + 1.5 Ti + 0.5 Nb	385 HV
S13	316L SS + 0.5 Ti + 1.5 Nb	327 HV
S14	316L SS + 1 Ti + 1 Nb	338 HV
S15	316L SS + 1.5 Ti + 0.5 Nb	350 HV

**Table 5:** Tensile test results of all formulations.

Alloy	Ultimate Tensile Strength (MPa)	Percentage Elongation (%)
S1	540.70	21.90
S2	358.91	10.41
S3	347.68	10.32
S4	331.19	10.21
S5	304.44	10.04
S6	449.84	17.02
S7	442.23	16.24
S8	429.04	17.57
S9	413.45	14.57
S10	409.23	14.15
S11	346.24	10.86
S12	354.27	10.21
S13	438.68	13.45
S14	414.23	13.81
S15	416.58	12.86

**Table 6:** Compressive yield strengths of all formulations.

Alloy	Composition	Yield Strength
S1	Pure 316L stainless steel	776
S2	316L SS + 0.25 B + 0.5 Ti	723
S3	316L SS + 0.25 B + 1 Ti	766
S4	316L SS + 0.25 B + 1.5 Ti	802
S5	316L SS + 0.25 B + 2 Ti	987
S6	316L SS + 0.25 B + 0.5 Nb	824
S7	316L SS + 0.25 B + 1 Nb	917
S8	316L SS + 0.25 B + 1.5 Nb	1102
S9	316L SS + 0.25 B + 2 Nb	1318
S10	316L SS + 0.25 B + 0.5 Ti + 1.5 Nb	850
S11	316L SS + 0.25 B + 1 Ti + 1 Nb	893
S12	316L SS + 0.25 B + 1.5 Ti + 0.5 Nb	747
S13	316L SS + 0.5 Ti + 1.5 Nb	1380
S14	316L SS + 1 Ti + 1 Nb	1408
S15	316L SS + 1.5 Ti + 0.5 Nb	1375

## Data Availability

Data sharing is not applicable for this paper.
